# Associations between the EQ-5D-5L and exacerbations of chronic obstructive pulmonary disease in the ETHOS trial

**DOI:** 10.1007/s11136-023-03582-z

**Published:** 2024-01-11

**Authors:** Dan Jackson, Martin Jenkins, Enrico de Nigris, Debasree Purkayastha, Mehul Patel, Mario Ouwens

**Affiliations:** 1 AstraZeneca, Cambridge, UK; 2 Formerly of AstraZeneca, Cambridge, UK; 3 AstraZeneca, Gothenburg, Sweden

**Keywords:** Chronic obstructive pulmonary disease (COPD), Quality of life, EuroQoL 5-dimension 5-level (EQ-5D-5L) questionnaire, Modeling

## Abstract

**Purpose:**

Exacerbations of chronic obstructive pulmonary disease (COPD) are associated with deteriorating health and health-related quality of life (HRQoL) among people with COPD during and after events. HRQoL data are key to evaluating treatment cost-effectiveness and informing reimbursement decisions in COPD. EuroQoL 5-dimension 5-level (EQ-5D-5L) utility scores, based on various HRQoL measures, are used in economic evaluations of pharmacotherapy. These analyses estimated associations between EQ-5D-5L utility scores and exacerbations (new and previous) in patients with moderate-to-very severe COPD.

**Methods:**

Longitudinal mixed models for repeated measures (MMRM), adjusted for time and treatment, were conducted using data from the ETHOS study (NCT02465567); models regressed EQ-5D-5L on current and past exacerbations that occurred during the study, adjusting for other patient reported outcomes and clinical factors.

**Results:**

Based on the simplest covariate adjusted model (adjusted for current exacerbations and number of previous exacerbations during the study), a current moderate exacerbation was associated with an EQ-5D-5L disutility of 0.055 (95% confidence interval: 0.048, 0.062) with an additional disutility of 0.035 (0.014, 0.055) if the exacerbation was severe. After resolving, each prior exacerbation was associated with a disutility that persisted for the remainder of the study (moderate exacerbation, 0.014 [0.011, 0.016]; further disutility for severe exacerbation, 0.011 [0.003, 0.018]).

**Conclusion:**

An EQ-5D-5L disutility of 0.090 was associated with a current severe exacerbation in ETHOS. Our findings suggest incorporating the effects of current, recently resolved, and cumulative exacerbations into economic models when estimating benefits and costs of COPD pharmacotherapy, as exacerbations have both acute and persistent effects.

**Supplementary Information:**

The online version contains supplementary material available at 10.1007/s11136-023-03582-z.

## Plain English Summary

People living with chronic obstructive pulmonary disease (COPD) experience symptoms such as coughing, difficulty breathing, and heavy mucus production. When COPD symptoms acutely worsen, the patient is said to have an “exacerbation”. Exacerbations can be “moderate”, resulting in the need for additional medicine, or “severe”, resulting in hospital admission or possibly death.

Exacerbations are often associated with lower quality of life. This study looked at how a patient’s quality of life is associated with the number and type of exacerbations they experience. Data from patients with moderate-to-very severe COPD who participated in a large clinical trial called ETHOS were used. The quality of a patient’s life was estimated using the EuroQoL 5-dimension 5-level survey, also called EQ-5D-5L. The EQ-5D-5L survey asks about aspects of daily living, such as ability to move, self-care, usual activities, pain/discomfort, and anxiety/depression. Survey answers are combined into a single score.

Findings from the study show how much lower quality of life is for patients living with COPD during and after an exacerbation. Lower quality of life is linked to the number and type of exacerbations patients experience. Measures of breathing difficulty do not necessarily fully explain the lower quality of life during and after exacerbations, indicating other factors may affect the quality of life of patients experiencing exacerbations. These findings may help healthcare providers further understand how much moderate or severe COPD exacerbations are related to a patient’s quality of life both during and after an exacerbation so appropriate courses of action can be taken.

## Introduction

Chronic obstructive pulmonary disease (COPD) is characterized by persistent airflow limitation and respiratory symptoms, including dyspnea, cough, and sputum production [1]. Despite being preventable and treatable [1], COPD is the third leading cause of death worldwide, accounting for approximately 6% of deaths in 2019 [2]. An acute worsening of COPD symptoms (i.e., an exacerbation [1]) may be defined as moderate when managed through primary care or outpatient services (oral corticosteroids and antibiotics) or severe if hospitalization and monitoring is required [3]. COPD exacerbations negatively impact lung function [4] and are detrimental to health-related quality of life (HRQoL) [5, 6], with the effects taking weeks to months to resolve [4].

One validated tool to assess HRQoL in patients with COPD is the EuroQoL 5-dimension 5-level (EQ-5D-5L) questionnaire [7, 8]. The EQ-5D-5L examines five dimensions of life (mobility, self-care, usual activities, pain/discomfort, and anxiety/depression) across five severity levels (no problems to extreme problems) [7]. EQ-5D-5L utility scores, anchored on 0 (death) and 1 (full health), can be used in economic evaluations [7]. EQ-5D-5L utility scores are based on HRQoL measures and exacerbations negatively affect HRQoL [4]. Previous studies have reported lower EQ-5D-3L utility scores in patients experiencing moderate or severe exacerbations [9–11] as well as associations between EQ-5D scores and the number of previous exacerbations [12, 13]. However, information on associations between exacerbations and EQ-5D-5L utility scores is still limited.

Mapping algorithms can predict EQ-5D utility scores in patients with COPD from HRQoL measurements obtained from the St. George’s Respiratory Questionnaire (SGRQ) [14]. Additionally, a small study demonstrated COPD severity and exacerbation frequency are associated with some patient-reported HRQoL measures based on visual analog scale (VAS) and time trade-off (TTO) assessments, with estimated reductions from 1 non-serious or serious exacerbation, respectively, of 0.037 and 0.090 for the VAS and 0.010 and 0.042 for TTO [6]. Moreover, a multivariate analysis of data from a UK COPD cohort study reported EQ-5D-5L scores were lowered by 0.082 and 0.143, respectively, in patients with any exacerbation or a severe exacerbation in the past 12 months [13]. However, further research is needed to expand our understanding of the magnitude of the EQ-5D-5L disutility associated with COPD exacerbations.

In the ETHOS study, patients with moderate-to-very severe COPD randomized to twice-daily triple inhaled corticosteroid/long-acting muscarinic antagonist/long-acting β_2_-agonist (ICS/LAMA/LABA) therapy with budesonide/glycopyrronium/formoterol fumarate dihydrate at two doses (320/14.4/10 μg [BGF 320] or 160/14.4/10 μg [BGF 160]) experienced lower annual moderate/severe exacerbation rates than patients randomized to dual therapy with the LAMA/LABA glycopyrronium/formoterol fumarate dihydrate 14.4/10 μg (GFF) or the ICS/LABA budesonide/formoterol fumarate dihydrate 320/10 μg (BFF) [15]. Additionally, improvements in HRQoL (measured by the SGRQ) favored BGF 320 and BGF 160 over GFF and BFF after 24 or 52 weeks of treatment [16].

The current analyses aimed to examine associations between COPD exacerbations and EQ-5D-5L utility scores in patients with moderate-to-very severe COPD from the ETHOS study using three questions. First, what is the magnitude of the association between exacerbations (both current and previous during the study) and EQ-5D-5L disutility? Second, to what extent do these associations exist conditionally based on lung function (measured by forced expiratory volume in 1 s [FEV_1_]) or HRQoL (measured by SGRQ). Finally, can EQ-5D-5L utility scores be predicted based on lung function, recent exacerbation history, and current exacerbation status? Although this work uses ETHOS data, the analyses examined general relationships between the EQ-5D-5L and exacerbations, rather than elucidating the benefits of BGF.

## Methods

### Population

These analyses utilized patients from the randomized, double-blind, parallel-group, Phase III, 52-week ETHOS study (NCT02465567), which assessed the efficacy and safety of BGF at two doses versus GFF or BFF in patients with COPD. Detailed descriptions of ETHOS and its population have been previously published [15, 17]. Briefly, patients were 40–80 years old, with symptomatic moderate-to-very severe COPD, had a post-bronchodilator ratio of FEV_1_ to the forced vital capacity of < 0.7 with a post-bronchodilator FEV_1_ of 25–65% of the predicted normal, and a history of ≥ 1 moderate/severe exacerbations (if FEV_1_ was < 50% of predicted normal) or ≥ 2 moderate exacerbations/ ≥ 1 severe exacerbation (if FEV_1_ was > 50% of predicted normal) in the year before screening [15]. Moderate exacerbations were defined as those leading to treatment with systemic glucocorticoids, antibiotics, or both for at least three days; severe exacerbations were those resulting in hospitalization (including to an emergency room or equivalent healthcare facility) or death.

Patients were randomized 1:1:1:1 to BGF 320/14.4/10 μg, BGF 160/14.4/10 μg, GFF 14.4/10 μg, or BFF 320/10 μg twice-daily over 52 weeks via a single metered dose inhaler [15]. These analyses used the modified intention-to-treat population (mITT; all patients with post-randomization data obtained before study drug discontinuation) of 8509 patients, of which 8498 patients had EQ-5D-5L, SGRQ, or FEV_1_ data; data from 11 patients did not contribute to the analyses. Data from 3316 patients with ≥ 1 post-randomization FEV_1_ assessment were available for analyses that included FEV_1_ assessment across multiple timepoints, with the majority being from the ETHOS pulmonary function sub-study (n = 3088 patients) [18].

## Model structure

Longitudinal models with EQ-5D-5L utility scores as the response were fitted to answer three questions. All models used mixed model for repeated measures (MMRM) with unstructured covariance matrices for EQ-5D-5L utility scores and were adjusted for time (visits; baseline and five post-baseline visits) and post-baseline treatment effects. The models included indicators for each post-baseline visit with interactions by treatment group, resulting in different average outcomes for each treatment group at each post-baseline visit. The same base model was used across questions, allowing for correlations among EQ-5D-5L utility scores within a patient [19]. Regression equations for each model are provided in Online resource 1. In Questions 1 and 2, the estimated population average effect of moderate and severe exacerbations was examined. Though adjusted for treatment, these analyses investigated associations between EQ-5D-5L and exacerbations rather than evaluating the benefits of BGF.

### Question 1: What is the magnitude of the association between exacerbations and EQ-5D-5L utility scores?

Three models explored Question 1 (Table 1). Model 1 included covariates for a current exacerbation (moderate or severe) and for a current severe exacerbation. The regression coefficient for the first covariate is the difference in EQ-5D-5L utility associated with a moderate exacerbation, and the regression coefficient for the second covariate is the additional difference associated with a severe exacerbation. The sum of these two coefficients is the difference associated with a severe exacerbation. We use the term “disutility” to refer to a difference with its sign reversed. Model 1 also included a covariate for the number of previous exacerbations (moderate or severe) during the study and a covariate for the number of previous severe exacerbations during the study. Models 2 and 2a included the same exacerbation covariates as Model 1, with additional baseline covariates (see Table 1). Model 2 used FEV_1_ percentage of predicted normal (FEV_1_% predicted) as a continuous variable; Model 2a used FEV_1_% predicted as a categorical variable (moderate COPD [reference category], severe COPD, very severe COPD). Severity based on FEV_1_% predicted was based on Global Initiative for Chronic Obstructive Lung Disease (GOLD) definitions (moderate FEV_1_% predicted, ≥ 50% and < 80%; severe FEV_1_% predicted, ≥ 30% and < 50%; very severe FEV_1_% predicted, < 30%) [1]. These models included data from 8498 patients with EQ-5D-5L utility scores from the mITT population.

**Table 1:** Covariates included across models for Questions 1–3

	Question 1			Question 2				Question 3
Covariate	Model 1	Model 2	Model 2a	Model 3	Model 4	Model 4a	Model 4b	Model 5
Current exacerbation (y/n)	✓	✓	✓	✓	✓	✓	✓	✓
Current severe exacerbation (y/n)	✓	✓	✓	✓				
Previous number of exacerbations during the study	✓	✓	✓	✓	✓	✓	✓	✓
Previous number of severe exacerbations during the study	✓	✓	✓	✓				
Baseline demographics and clinical characteristics^a^		✓	✓	✓	✓	✓	✓	
Baseline FEV_1_% predicted (continuous)		✓			✓			
Baseline FEV_1_% predicted (categorical)^b^			✓	✓		✓	✓	
SGRQ total score and SGRQ squared total score by visit				✓			✓	
Current FEV_1_% predicted (continuous)					✓			
Current FEV_1_% predicted (categorical)^b^						✓	✓	✓
Current exacerbation by current FEV_1_% predicted severity (categorical)^b^ interaction								✓
Post-baseline treatment effects and visit	✓	✓	✓	✓	✓	✓	✓	✓

^a^Includes age, sex, geographic location, log eosinophil count, CAT score, ICS use (y/n), number of COPD exacerbations in the previous year before entry into the study, and severe COPD exacerbation in previous year before entry into the study (y/n)

^b^Moderate COPD (reference category) vs severe or very severe COPD. Moderate FEV_1_% predicted was defined as ≥ 50% and < 80%; severe FEV_1_% predicted was defined as ≥ 30% and < 50%; very severe FEV_1_% predicted was defined as < 30%

*CAT* COPD Assessment Test, *COPD* chronic obstructive pulmonary disease, *FEV*_*1*_*% predicted* forced expiratory volume in 1 s percentage of predicted normal value, *ICS* inhaled corticosteroids, *SGRQ* St. George’s Respiratory Questionnaire

### Question 2: To what extent is the association between exacerbations and EQ-5D-5L utility scores explained by the associations between exacerbations and either SGRQ or FEV_1_?

Four models explored Question 2 (Table 1). Model 3 included the same covariates as Model 2a, with the addition of SGRQ total score by visit and its squared value by visit as time-varying covariates, as SGRQ and its squared value are important predictors of utility [14]. Model 3 included data from the same 8498 patients used in Models 1 and 2.

Models 4 and 4a included current exacerbations (moderate or severe) and the number of previous exacerbations (moderate or severe) during the study (describing the association of EQ-5D-5L with an exacerbation), all baseline variables included in Models 2 and 2a, respectively, and current FEV_1_ severity as continuous (Model 4; based on post-bronchodilator FEV_1_% predicted) or categorical (Model 4a; moderate COPD [reference category], severe, and very severe COPD) covariates. Models 4 and 4a examined whether associations between EQ-5D-5L utility scores and exacerbations were retained after adjusting for current FEV_1_ severity or if they existed conditionally based on FEV_1_ severity. Model 4b included the same covariates as Model 4a, with the addition of SGRQ total score by visit and its squared value by visit as time-varying covariates to examine whether associations between EQ-5D-5L utility scores and exacerbations were retained after adjusting for current FEV_1_ severity and SGRQ total score. Data from 3316 patients with EQ-5D-5L utility scores and FEV_1_ information were used in Models 4, 4a, and 4b. As there were fewer severe exacerbations than moderate exacerbations in this population, Models 4, 4a, and 4b did not include covariates for current severe exacerbations or the number of previous severe exacerbations during the study. Hence, covariate effects for associations between exacerbations and EQ-5D-5L utility scores included exacerbations independent of severity.

### Question 3: What would be the predicted EQ-5D-5L utility score for a given disease severity and exacerbation status (current and previous)?

Model 5 (Table 1) explored Question 3 and included visit, post-baseline treatment effects, number of previous exacerbations during the study, and the interaction between a current exacerbation (yes/no) and current FEV_1_ severity (categorical: moderate COPD [reference category], severe, and very severe COPD). The model allowed for different EQ-5D-5L utility scores for each current FEV_1_ level and for different disutilities of current exacerbations by FEV_1_ level. The model assumed the association between the cumulative number of previous exacerbations during the study and EQ-5D-5L utility score was the same across FEV_1_ severity levels, reflecting the disutility of HRQoL during exacerbations and after they have subsided, regardless of FEV_1_ severity level. As for Models 4, 4a, and 4b, Model 5 used data from 3316 patients with EQ-5D-5L utility scores and FEV_1_ information and did not include covariates for current severe exacerbations or the number of previous severe exacerbations during the study. The objective of Question 3 differed from Question 1 in that Question 3 aimed to predict individual EQ-5D-5L scores from exacerbation status and FEV_1_, as would be useful in a health economic model, rather than in investigating population level associations.

### Assumptions and handling of missing data

The analyses assumed missing data (EQ-5D-5L utility scores and SGRQ scores) were missing at random (MAR; i.e., the probability of missing data does not depend on unobserved data). A sensitivity analysis using a data not missing at random (MNAR) assumption was completed to support Model 5 (see online resources for full details) due to concerns that EQ-5D-5L and SGRQ scores are more likely to be missing for patients with poorer HRQoL, which would result in positive bias (i.e., an upward shift) in the predicted baseline covariate identified in the model.

## Results

### Population characteristics

Across ETHOS treatment groups, mean age was 64.7 years, 59.7% of patients were male, and mean post-rescue medication FEV_1_% predicted was 43.4% in the mITT population (Online resource 2). In the 12 months preceding the study, patients in each treatment group averaged 1.7 exacerbations, with approximately 21% of patients having ≥ 1 severe exacerbation. Across treatment groups, 47.8–50.9% of patients had ≥ 1 exacerbation during the study.

### Question 1: What is the magnitude of the association between exacerbations and EQ-5D-5L utility scores?

**Fig. 1:**
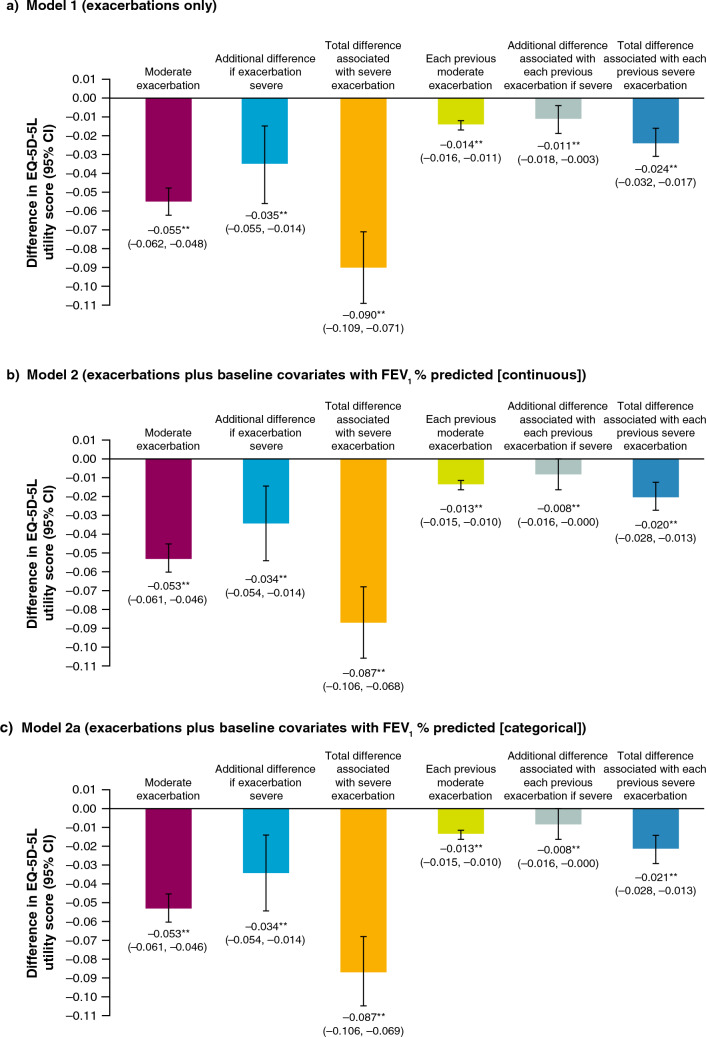
Coefficients of exacerbation variables for EQ-5D-5L utility scores (Question 1: Models 1, 2, and 2a)^a,b^. Note: EQ-5D-5L disutility score for FEV_1_ (continuous) represents the disutility associated with a 1% reduction in FEV_1_ Error bars indicate the 95% CI Wald p-values; *p < 0.05, **p < 0.01 Values for the total effect of a current or previous severe exacerbation may not equal the sum of the disutilities associated with a moderate exacerbation and the additional disutility if the exacerbation is severe due to rounding. ^a^Models include covariates as outlined in Table 1. ^b^Based on 8498 patients *CI* confidence interval, *EQ-5D-5L* EuroQoL 5-dimension 5-level, *FEV*_*1*_*% predicted* forced expiratory volume in 1 s percentage of predicted normal value

When only exacerbation covariates were considered, an EQ-5D-5L disutility of 0.055 was associated with a current moderate exacerbation during a visit compared with a visit without a current exacerbation (Fig. 1a [Model 1]). Severe exacerbations were associated with an additional EQ-5D-5L disutility of 0.035, totalling a disutility of 0.090. Each previous moderate exacerbation during the study was associated with an EQ-5D-5L disutility of 0.014, with an additional disutility of 0.011 for each previous severe exacerbation during the study. Accordingly, the total disutility of a previous severe exacerbation during the study was 0.024 (0.017, 0.032). These coefficients indicate both immediate (disutility of 0.055 for an acute moderate event) and lasting (disutility of 0.014 remaining after moderate event resolution) associations of exacerbations on EQ-5D-5L utility scores. EQ-5D-5L disutilities persisted and were similar when including baseline covariates in the models (Fig. 1b and 1c [Models 2 and 2a]).

### Question 2: To what extent is the association between exacerbations and EQ-5D-5L utility scores explained by the associations between exacerbations and either SGRQ or FEV_1_?

When SGRQ score was included as a covariate (Model 3), in addition to the covariates included in Model 2a, disutilities (95% confidence interval [CI]) associated with SGRQ and SGRQ squared values were 0.0027 (0.0023, 0.0030) and 0.000035 (0.000031, 0.000039) per unit increase in SGRQ and SGRQ squared, respectively, indicating larger SGRQ scores are associated with lower EQ-5D-5L utility. In this model, a current moderate exacerbation was associated with a disutility (95% CI) of 0.012 (0.005, 0.019), with an additional disutility of 0.021 (0.002, 0.039) if the exacerbation was severe, totalling 0.033 (0.015, 0.050) for a current severe exacerbation. Notably, EQ-5D-5L disutilities (95% CI) associated with previous exacerbations during the study were not observed when including SGRQ in the model (disutility for each previous moderate exacerbation: 0.001 [–0.001, 0.004]; additional disutility if severe: 0.003 [–0.004, 0.009]; total disutility for severe exacerbation: 0.004 [–0.002, 0.011]). Using a similar model, where current continuous post-bronchodilator FEV_1_% predicted was used as a covariate rather than a baseline categorical variable, comparable results were observed but, for brevity, are not reported. Together, these results suggest SGRQ and its squared value are good predictors that may partially account for associations between EQ-5D-5L utility scores and exacerbations because the magnitude of the associations was smaller when included in the model.

**Fig. 2:**
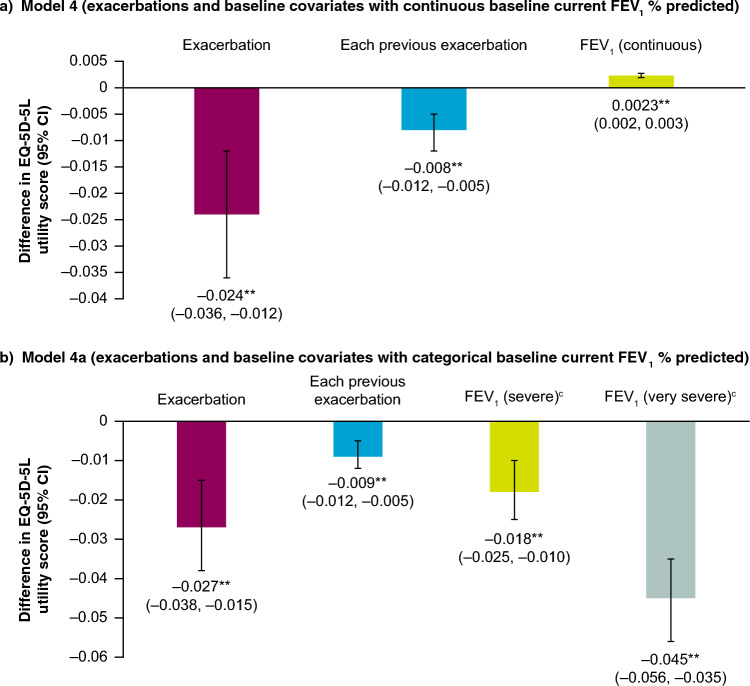
Coefficients for associations of exacerbations with EQ-5D-5L utility scores when accounting for baseline covariates with a) continuous FEV_1_ (Question 2: Model 4) and b) FEV_1_ severity (Question 2: Model 4a)^a,b^. Note: EQ-5D-5L disutility score for FEV_1_ (continuous) represents the disutility associated with a 1% reduction in FEV_1_ Wald p-values; **p < 0.01. ^a^Models include covariates as described in Table 1. ^b^Based on 3316 patients. ^c^Moderate COPD (reference category). Moderate FEV_1_% predicted was defined as ≥ 50% and < 80%; severe FEV_1_% predicted was defined as ≥ 30% and < 50%; very severe FEV_1_% predicted was defined as < 30% *CI* confidence interval, *EQ-5D-5L* EuroQoL 5-dimension 5-level, *FEV*_*1*_ forced expiratory volume in 1 s, *FEV*_*1*_*% predicted* forced expiratory volume in 1 s percentage of predicted normal value

When current FEV_1_ severity was added as a covariate, associations between EQ-5D-5L utility scores and current exacerbations and the number of previous exacerbations during the study were retained (Fig. 2), suggesting FEV_1_ does not fully account for associations between exacerbations and EQ-5D-5L utility scores. In the model with continuous FEV_1_ as a covariate (Model 4), current exacerbations were associated with an EQ-5D-5L disutility of 0.024, with an additional disutility of 0.008 for each previous exacerbation during the study (Fig. 2a). In the model with categorical FEV_1_ as a covariate (Model 4a), severe or very severe current FEV_1_ was associated with EQ-5D-5L disutilities of 0.018 or 0.045, respectively, compared with moderate current FEV_1_ severity. Current exacerbations were associated with an EQ-5D-5L disutility of 0.027, with an additional disutility of 0.009 for each previous exacerbation (Fig. 2b). When SGRQ was included as a covariate (Model 4b), in addition to the covariates included in Model 4a, disutilities (95% CI) of 0.0026 (0.019, 0.0033) and 0.000032 (0.000025, 0.000039) were associated with an increase in SGRQ and SGRQ squared per unit, respectively. Notably, in this model, associations between a current exacerbation (disutility [95% CI]: –0.004 [–0.015, 0.007]) and each previous exacerbation during the study (0.002 [–0.001, 0.005]) were not observed when SGRQ was included. Disutilities associated with severe and very severe FEV_1_, respectively, were 0.004 (–0.003, 0.011) and 0.012 (0.002, 0.022). These results indicate the association of a current exacerbation and EQ-5D-5L is not fully accounted for by the SGRQ (Model 3) or by FEV_1_ severity (Models 4 and 4a) alone, but may be fully accounted for when both SGRQ and FEV_1_ severity are included (Model 4b).

### Question 3: What is the predicted EQ-5D-5L utility score for a given disease severity and exacerbation status (current and previous)?

Across all visits and treatment groups, Model 5 predicted that the EQ-5D-5L utility score among patients with moderate COPD and no current exacerbations or previous exacerbations during the study was 0.739 (Table 2), with EQ-5D-5L disutilities of 0.016 for severe COPD and 0.048 for very severe COPD. A current exacerbation was associated with an EQ-5D-5L disutility of 0.015, with additional disutilities associated with a current exacerbation based on COPD states: 0.009 (severe COPD) or 0.023 (very severe COPD). Each previous exacerbation was associated with an EQ-5D-5L disutility of 0.009, irrespective of exacerbation severity. Results from the MNAR sensitivity analysis were similar to those from the MAR model (**Online resource 3**).

**Table 2:** Predictors of EQ-5D-5L based on FEV_1_ levels and exacerbation events (Question 3; Model 5)^a,b^

Variable	Coefficient (95% CI)
Predicted baseline EQ-5D-5L utility score for patients with currently moderate COPD with no current or previous exacerbation	0.739 (0.731, 0.748)
*Covariate adjustment from baseline (currently moderate COPD severity with no current or previous exacerbation during the ETHOS trial) in EQ-5D-5L utility score*	
Severe COPD	–0.016 (–0.024, –0.009)**
Very severe COPD	–0.048 (–0.058, –0.038)**
Current exacerbation	–0.015 (–0.046, 0.017)
Current exacerbation, additional effect if severe COPD	–0.009 (–0.044, 0.026)
Current exacerbation, additional effect if very severe COPD	–0.023 (–0.060, 0.014)
Previous exacerbations	–0.009 (–0.012, –0.005)**

Data represent estimates (95% CI)

Wald p-values; **p < 0.01

^a^Model includes covariates as described in Table 1

^b^Based on 3316 patients

*CI* confidence interval, *COPD* chronic obstructive pulmonary disease, *EQ-5D-5L* EuroQoL 5-dimension 5-level, *FEV*_*1*_ forced expiratory volume in 1 second

To demonstrate the model, consider hypothetical ‘Patient A’ from the ETHOS study, who has very severe COPD, experienced one previous exacerbation during the study, and is currently experiencing an exacerbation. The model predicts the base case (moderate COPD with no exacerbations experienced during the study) EQ-5D-5L utility score is 0.739. The predicted EQ-5D-5L disutility for ‘Patient A’ would be: 0.048 for having very severe COPD, 0.009 for having one previous exacerbation during the study, and 0.015 + 0.023 for having a current exacerbation and having very severe COPD, respectively. Therefore, ‘Patient A’ would have an EQ-5D-5L utility score of 0.644 (0.739 base case − 0.095 disutility).

## Discussion

These analyses considered three questions to examine associations between COPD exacerbations and EQ-5D-5L utility scores using ETHOS study data in distinct statistical models with differing covariates. Question 1 quantified the association between exacerbations and EQ-5D-5L utility scores (Model 1) and assessed if this association was robust to the inclusion of baseline demographics, clinical characteristics, and FEV_1_% predicted (Model 2 and 2a). Question 2 assessed if the observed associations were explained by covariates for lung function and HRQoL (Models 3–4b). Finally, in a change of objective, Question 3 aimed to predict EQ-5D-5L utility scores given current and previous exacerbation status and disease severity (Model 5).

Exacerbations of COPD were associated with acute (based on current exacerbations) and continuing (based on previous exacerbations during the study) EQ-5D-5L disutility in patients with moderate-to-very severe COPD. Severe exacerbations and more severe COPD (as measured by FEV_1_% predicted) were associated with further EQ-5D-5L disutility. Measures of lung function (FEV_1_ severity) or patient-reported measures of breathing problems (SGRQ total scores) separately did not fully explain the EQ-5D-5L disutility associated with exacerbations, but together they may. Lastly, Model 5 predicted EQ-5D-5L utility scores in patients with COPD and EQ-5D-5L disutility during or after exacerbations based on varying FEV_1_ severity and exacerbation history.

Our findings quantify the extent to which EQ-5D-5L disutility is associated with current exacerbations and their severity in patients with COPD. Importantly, associations of exacerbations with EQ-5D-5L utility scores remain after resolution of an exacerbation, as indicated by further EQ-5D-5L disutility with each previous exacerbation during the study. As such, it may be insufficient to consider current exacerbations and exacerbation history in isolation when estimating EQ-5D-5L disutility because the impact of current exacerbations on HRQoL may depend on exacerbation history, though not considered in previously published cost-effectiveness models for COPD [20–23]. In Markov models, patients sojourn in disease severity states, with patients developing moderate or severe exacerbations according to risks associated with each state. Costs and quality of life scores attributed to exacerbations and severity states are compared between treatment and control arms to determine incremental costs per quality-adjusted life year. The current analyses provide important new insights by demonstrating additive effects of previous exacerbations on EQ-5D-5L disutility should be considered in future evaluations. Model 5 showed the effects of exacerbations on EQ-5D-5L disutility were greater in patients with poorer lung function (as indicated by greater FEV_1_ severity). These findings suggest effective maintenance pharmacotherapies could preserve EQ-5D-5L utility scores in patients with COPD. Overall, these results suggest exacerbations would be associated with HRQoL disutility in patients with moderate-to-very severe COPD, even if other assessments were used. Therefore, the current results could help to improve policy and decision making through health economics evaluations.

These results are consistent with previous reports. A previous meta-analysis reported that moderate and severe exacerbations were associated with lower EQ-5D-5L, with severe exacerbations having a greater impact than moderate exacerbations [24]. In an analysis of the overall population (n = 18,746) and the population of patients with ≥ 1 prior severe exacerbations (n = 4483), an increase in moderate and severe exacerbations of one per year was associated with EQ-5D-5L index score disutilities of 0.02 and 0.03, respectively [24]. Our results are also consistent with a previous publication by Rutten-van Molken et al. that assessed the effect of COPD health status on HRQoL [6], although there are methodological differences between these analyses that limit study comparability. Rutten-van Molken et al. assessed the effect of COPD health status (based on COPD severity, non-serious exacerbations, and serious exacerbations) on HRQoL in 239 patients with COPD, as measured by VAS and TTO (as opposed to EQ-5D-5L) [6]. Their analysis demonstrated consistently lower VAS and TTO values over a one-year period for more severe COPD (mild versus severe) and as the number of exacerbations increased. For non-serious and serious exacerbations, respectively, estimated VAS disutilities were 0.037 and 0.090 and TTO disutilities were 0.010 and 0.042. So, TTO disutilities (another preference based method) were smaller in magnitude than disutilities observed in the current study (0.055 for current non-severe exacerbations; 0.090 for current severe exacerbations). However, methodologic issues make direct comparisons between these studies difficult. Rutten-van Molken et al. examined VAS and TTO values using mild COPD as the base case and enrolled patients with and without exacerbation histories [6], whereas our analysis used moderate COPD as the base case and included only patients with an exacerbation history before study enrolment. Furthermore, a benefit of the current analysis is that the EQ-5D-5L is preference based, relying on weighted estimates for each item from a large sample and for a representative sample of the general public. The EQ-5D-5L is preferred for economic evaluations for decision making over direct methods, such as VAS [25].

The strengths of this analysis include using data from large cohorts (n = 8498 or n = 3316 depending on the model), allowing for robust findings. Furthermore, the predictive model examined the joint effects of clinical dynamics (lung function) and clinical events (exacerbations) to show strong associations of exacerbations with EQ-5D-5L disutility and presumably HRQoL.

This analysis was limited by the 52-week ETHOS study duration, as the effect of exacerbations on EQ-5D-5L utility scores beyond one year of follow-up could not be evaluated. Therefore, longer-term associations between exacerbations and EQ-5D-5L utility scores could not be extrapolated. The current analysis also did not include populations beyond the ETHOS study, for example patients with mild COPD and no recent exacerbation history. Furthermore, the analysis did not account for a boundary effect, as the modeling assumptions allowed maximum EQ-5D-5L utility score to exceed 1 (a value outside the instrument’s anchored range). Finally, though the current findings demonstrate associations between exacerbations and EQ-5D-5L disutility, these analyses do not attempt to infer causation. Further investigations may be useful to assess the impact of additional covariates, infer causation, or validate the observed associations between exacerbations and EQ-5D-5L utility scores.

## Conclusions

Statistical modeling indicated that current and previous exacerbations of COPD were associated with disutility on the EQ-5D-5L, which measures various aspects of HRQoL [7], in patients with moderate-to-very severe COPD from the ETHOS study. Severe exacerbations and more severe disease were associated with further EQ-5D-5L disutility. Inclusion of lung function or patient-reported measures of breathing problems alone may not fully explain the EQ-5D-5L disutility associated with exacerbations, but together they may. Importantly, the EQ-5D-5L disutility associated with a current severe exacerbation from Models 1, 2 and 2a was larger than that observed in other economic evaluations [6]. However, these findings require confirmation after accounting for COPD severity, for example by fitting a model similar to Model 5 that distinguishes between the associations of severe and non-severe exacerbations. To adequately identify this type of model, data from a sufficient number of patients with known current COPD severity and a sufficiently high rate of severe exacerbations would be required. Overall, these results suggest health economic evaluations should consider both current and previous exacerbation status, including the severity and number of exacerbations, on EQ-5D-5L when estimating the benefits of COPD pharmacotherapy, rather than just mapping from SGRQ or modeling the progression of disease severity and lung function decline. Predictive modeling of EQ-5D-5L utility using lung function and exacerbation data may help healthcare professionals to understand the HRQoL of patients with moderate-to-very severe COPD.

### Supplementary Information

Below is the link to the electronic supplementary material.

Supplementary file1 (PDF 401 KB)

## Data Availability

Not applicable.
