# Supplementary material for: Associations between the EQ-5D-5L and exacerbations of chronic obstructive pulmonary disease in the ETHOS trial
Source: Qual Life Res. 2024 Jan 11;33(4):1029–39. doi: 10.1007/s11136-023-03582-z (PMC10973049; doi:10.1007/s11136-023-03582-z)
Supplement: Supplementary file 1 — Supplementary file1 (PDF 401 KB) [file 11136_2023_3582_MOESM1_ESM.pdf]

# ONLINE RESOURCES

## Associations between the EQ-5D-5L and exacerbations of chronic obstructive pulmonary disease in the ETHOS trial

**Journal:** *Qual Life Res*

Dan Jackson,[1] Martin Jenkins,[1] Enrico de Nigris,[2] Debasree Purkayastha,[1] Mehul Patel,[1] Mario Ouwens[3]

[1]*AstraZeneca, Cambridge, UK;* [2]*Formerly of AstraZeneca, Cambridge, UK;* [3]*AstraZeneca, Gothenburg, Sweden*

**Correspondence to:** Dan Jackson, Academy House, 136 Hills Rd, Cambridge CB2 8PA, UK; daniel.jackson1@astrazeneca.com

### Sensitivity analysis methods

The sensitivity analysis was based on a data not missing at random assumption. It is possible that EuroQoL 5-dimension 5-level (EQ-5D-5L) and St. George's Respiratory Questionnaire (SGRQ) scores are more likely to be missing for patients with poorer health-related quality of life, so multiple imputation was used to impute scores at patient-visits where values were missing. This imputation model included all variables from Models 1, 2, and 3 (including the outcomes) as well as patient-visit EQ-5D-5L utility scores. Predictive mean matching was used for continuous outcomes to ensure valid values were imputed.

Overall, 100 imputed datasets were created. The 5% and 95% EQ-5D-5L utility scores and SGRQ scores across the 100 missing values were singly imputed as a sensitivity analysis. SGRQ scores were not used in the analysis. This sensitivity analysis did not enforce the relationship between SGRQ total score and the squared valued of the SGRQ total score (the squared value was imputed as another variable), and it did not consider the interaction between exacerbation and disease severity. Due to the complexity of the model, only baseline

and information from the same patient-visit were used in the imputation model. Multiple imputation creates complete datasets for all variables, but only EQ-5D-5L and SGRQ scores were then singly imputed using the 5% and 95% quantiles across the 100 imputed datasets to capture the possibility that missing values for the two patients reported outcomes may be lower than assumed when assuming missing at random. Current forced expiratory volume in 1 second ($FEV_1$) severity was not imputed so that information from patient visits where this is missing was not recovered in the sensitivity analysis. Current $FEV_1$ severity was not imputed.

**Online resource 1** Listing of regression coefficients used across models

| Model 1[a] | | | | |
|---|---|---|---|---|
| | Lower 95% CI | Estimate | Upper 95% CI | Data reported in: |
| (Intercept) | 0.705 | 0.710 | 0.714 | |
| Visit_two | 0.030 | 0.037 | 0.044 | |
| Visit_three | 0.036 | 0.044 | 0.051 | |
| Visit_four | 0.042 | 0.049 | 0.057 | |
| Visit_five | 0.036 | 0.044 | 0.052 | |
| Visit_six | 0.042 | 0.050 | 0.059 | |
| Moderate exacerbation | –0.062 | –0.055 | –0.048 | Figure 1a |
| Severe exacerbation | –0.055 | –0.035 | –0.014 | Figure 1a |
| Previous moderate exacerbation | –0.016 | –0.014 | –0.011 | Figure 1a |
| Previous severe exacerbation | –0.018 | –0.011 | –0.003 | Figure 1a |
| Visit_two: 160 µg BGF | –0.006 | 0.003 | 0.012 | |
| Visit_two: 320 µg BGF | –0.002 | 0.008 | 0.017 | |
| Visit_two: GFF | –0.005 | 0.005 | 0.014 | |
| Visit_three: 160 µg BGF | –0.010 | 0.000 | 0.010 | |
| Visit_three: 320 µg BGF | –0.004 | 0.006 | 0.016 | |
| Visit_three: GFF | –0.007 | 0.003 | 0.013 | |
| Visit_four: 160 µg BGF | –0.013 | –0.003 | 0.007 | |
| Visit_four: 320 µg BGF | –0.004 | 0.006 | 0.016 | |
| Visit_four: GFF | –0.006 | 0.004 | 0.015 | |
| Visit_five: 160 µg BGF | –0.005 | 0.006 | 0.017 | |
| Visit_five: 320 µg BGF | –0.009 | 0.002 | 0.013 | |
| Visit_five: GFF | 0.000 | 0.011 | 0.022 | |
| Visit_six: 160 µg BGF | –0.010 | 0.001 | 0.012 | |
| Visit_six: 320 µg BGF | –0.005 | 0.006 | 0.017 | |
| Visit_six: GFF | –0.002 | 0.009 | 0.020 | |

| Model 2[a,b,c] | | | | |
|---|---|---|---|---|
| | Lower 95% CI | Estimate | Upper 95% CI | Data reported in: |
| (Intercept) | 0.824 | 0.862 | 0.901 | |
| Visit_two | 0.030 | 0.036 | 0.043 | |
| Visit_three | 0.035 | 0.043 | 0.050 | |
| Visit_four | 0.040 | 0.048 | 0.055 | |
| Visit_five | 0.034 | 0.042 | 0.050 | |
| Visit_six | 0.039 | 0.047 | 0.056 | |
| Moderate exacerbation | –0.061 | –0.053 | –0.046 | Figure 1b |
| Severe exacerbation | –0.054 | –0.034 | –0.014 | Figure 1b |
| Previous moderate exacerbation | –0.015 | –0.013 | –0.010 | Figure 1b |
| Previous severe exacerbation | –0.016 | –0.008 | 0.000 | Figure 1b |
| Age (years) | –0.0006 | –0.0002 | 0.0002 | |
| Male | –0.003 | 0.003 | 0.009 | |
| US & Canada | –0.002 | 0.006 | 0.013 | |
| Asia | 0.079 | 0.093 | 0.106 | |
| Australia & New Zealand & South Africa | 0.031 | 0.045 | 0.059 | |
| Eastern Europe | 0.050 | 0.061 | 0.071 | |
| Mexico & Central America & South America | 0.018 | 0.027 | 0.037 | |
| LOG eosinophil count | 0.004 | 0.009 | 0.014 | |
| Number of exacerbations previous year | –0.006 | –0.002 | 0.001 | |
| $FEV_1$ continuous | 0.001 | 0.001 | 0.001 | |
| CAT score | –0.013 | –0.013 | –0.012 | |
| ICS use | –0.007 | –0.001 | 0.006 | |
| Severe exacerbation previous year | –0.030 | –0.023 | –0.015 | |
| Visit_two: 160 µg BGF | –0.006 | 0.003 | 0.012 | |
| Visit_two: 320 µg BGF | 0.000 | 0.009 | 0.018 | |
| Visit_two: GFF | –0.005 | 0.004 | 0.013 | |
| Visit_three: 160 µg BGF | –0.009 | 0.001 | 0.011 | |
| Visit_three: 320 µg BGF | –0.002 | 0.008 | 0.018 | |

| | | | | |
|---|---|---|---|---|
| Visit_three: GFF | –0.008 | 0.002 | 0.012 | |
| Visit_four: 160 µg BGF | –0.012 | –0.002 | 0.008 | |
| Visit_four: 320 µg BGF | –0.002 | 0.008 | 0.018 | |
| Visit_four: GFF | –0.007 | 0.004 | 0.014 | |
| Visit_five: 160 µg BGF | –0.005 | 0.006 | 0.017 | |
| Visit_five: 320 µg BGF | –0.006 | 0.004 | 0.015 | |
| Visit_five: GFF | –0.001 | 0.010 | 0.021 | |
| Visit_six: 160 µg BGF | –0.009 | 0.002 | 0.013 | |
| Visit_six: 320 µg BGF | –0.003 | 0.009 | 0.020 | |
| Visit_six: GFF | –0.003 | 0.008 | 0.020 | |

| Model 2a[a,b,c,d] | | | | |
|---|---|---|---|---|
| | Lower 95% CI | Estimate | Upper 95% CI | Data reported in: |
| (Intercept) | 0.891 | 0.929 | 0.967 | |
| Visit_two | 0.030 | 0.036 | 0.043 | |
| Visit_three | 0.035 | 0.043 | 0.050 | |
| Visit_four | 0.040 | 0.048 | 0.055 | |
| Visit_five | 0.034 | 0.042 | 0.050 | |
| Visit_six | 0.039 | 0.047 | 0.056 | |
| Moderate exacerbation | –0.061 | –0.053 | –0.046 | Figure 1c |
| Severe exacerbation | –0.054 | –0.034 | –0.014 | Figure 1c |
| Previous moderate exacerbation | –0.015 | –0.013 | –0.010 | Figure 1c |
| Previous severe exacerbation | –0.016 | –0.008 | 0.000 | Figure 1c |
| Age (years) | –0.0006 | –0.0002 | 0.0002 | |
| Male | –0.003 | 0.003 | 0.009 | |
| US & Canada | –0.002 | 0.006 | 0.013 | |
| Asia | 0.079 | 0.092 | 0.105 | |
| Australia & New Zealand & South Africa | 0.031 | 0.045 | 0.059 | |
| Eastern Europe | 0.050 | 0.060 | 0.071 | |
| Mexico & Central America & South America | 0.018 | 0.027 | 0.037 | |

| | | | | |
|---|---|---|---|---|
| LOG eosinophil count | 0.004 | 0.009 | 0.014 | |
| Number of exacerbations previous year | –0.006 | –0.002 | 0.002 | |
| Severe COPD | –0.024 | –0.018 | –0.011 | |
| Very severe COPD | –0.050 | –0.040 | –0.029 | |
| CAT score | –0.013 | –0.013 | –0.012 | |
| ICS use | –0.007 | –0.001 | 0.006 | |
| Severe exacerbation previous year | –0.030 | –0.023 | –0.016 | |
| Visit_two: 160 µg BGF | –0.006 | 0.003 | 0.012 | |
| Visit_two: 320 µg BGF | 0.000 | 0.009 | 0.018 | |
| Visit_two: GFF | –0.005 | 0.004 | 0.013 | |
| Visit_three: 160 µg BGF | –0.009 | 0.001 | 0.010 | |
| Visit_three: 320 µg BGF | –0.002 | 0.008 | 0.018 | |
| Visit_three: GFF | –0.008 | 0.002 | 0.012 | |
| Visit_four: 160 µg BGF | –0.012 | –0.002 | 0.008 | |
| Visit_four: 320 µg BGF | –0.002 | 0.008 | 0.018 | |
| Visit_four: GFF | –0.007 | 0.004 | 0.014 | |
| Visit_five: 160 µg BGF | –0.005 | 0.006 | 0.017 | |
| Visit_five: 320 µg BGF | –0.006 | 0.004 | 0.015 | |
| Visit_five: GFF | –0.001 | 0.010 | 0.021 | |
| Visit_six: 160 µg BGF | –0.009 | 0.002 | 0.013 | |
| Visit_six: 320 µg BGF | –0.003 | 0.009 | 0.020 | |
| Visit_six: GFF | –0.003 | 0.008 | 0.020 | |

| Model 3[a,c,d] | | | | |
|---|---|---|---|---|
| | Lower 95% CI | Estimate | Upper 95% CI | Data reported in: |
| (Intercept) | 0.955 | 0.986 | 1.017 | |
| Visit_two | 0.000 | 0.006 | 0.012 | |
| Visit_three | 0.000 | 0.006 | 0.012 | |
| Visit_four | 0.003 | 0.010 | 0.016 | |
| Visit_five | –0.006 | 0.001 | 0.008 | |

| | | | | |
|---|---|---|---|---|
| Visit_six | –0.002 | 0.005 | 0.012 | |
| Moderate exacerbation | –0.019 | –0.012 | –0.005 | Results text (as disutility) |
| Severe exacerbation | –0.039 | –0.021 | –0.002 | Results text (as disutility) |
| Previous moderate exacerbation | –0.004 | –0.001 | 0.001 | Results text (as disutility) |
| Previous severe exacerbation | –0.009 | –0.003 | 0.004 | Results text (as disutility) |
| Age (years) | –0.0004 | –0.0001 | 0.0002 | |
| Male | 0.001 | 0.006 | 0.011 | |
| US & Canada | 0.000 | 0.006 | 0.013 | |
| Asia | 0.040 | 0.051 | 0.062 | |
| Australia & New Zealand & South Africa | 0.019 | 0.030 | 0.041 | |
| Eastern Europe | 0.027 | 0.036 | 0.044 | |
| Mexico & Central America & South America | 0.006 | 0.014 | 0.021 | |
| LOG eosinophil count | 0.002 | 0.006 | 0.010 | |
| Number of exacerbations previous year | –0.003 | 0.000 | 0.003 | |
| Severe COPD | –0.005 | 0.000 | 0.006 | |
| Very severe COPD | –0.010 | –0.002 | 0.007 | |
| CAT score | –0.005 | –0.004 | –0.004 | |
| ICS use | –0.005 | 0.000 | 0.006 | |
| Severe exacerbation previous year | –0.018 | –0.013 | –0.007 | |
| SGRQ total | –0.0030 | –0.0027 | –0.0023 | Results text (as disutility) |
| SGRQ total squared | –0.000039 | –0.000035 | –0.000031 | Results text (as disutility) |
| Visit_two: 160 μg BGF | –0.011 | –0.003 | 0.005 | |
| Visit_two: 320 μg BGF | –0.006 | 0.002 | 0.010 | |
| Visit_two: GFF | –0.004 | 0.004 | 0.012 | |
| Visit_three: 160 μg BGF | –0.014 | –0.005 | 0.003 | |
| Visit_three: 320 μg BGF | –0.009 | –0.001 | 0.008 | |
| Visit_three: GFF | –0.006 | 0.003 | 0.011 | |
| Visit_four: 160 μg BGF | –0.017 | –0.008 | 0.000 | |
| Visit_four: 320 μg BGF | –0.009 | 0.000 | 0.008 | |

| | | | | |
|---|---|---|---|---|
| Visit_four: GFF | –0.007 | 0.001 | 0.010 | |
| Visit_five: 160 µg BGF | –0.008 | 0.001 | 0.010 | |
| Visit_five: 320 µg BGF | –0.009 | 0.000 | 0.009 | |
| Visit_five: GFF | 0.000 | 0.009 | 0.018 | |
| Visit_six: 160 µg BGF | –0.012 | –0.002 | 0.007 | |
| Visit_six: 320 µg BGF | –0.007 | 0.002 | 0.011 | |
| Visit_six: GFF | 0.000 | 0.010 | 0.019 | |

| Model 4[a,c] | | | | |
|---|---|---|---|---|
| | Lower 95% CI | Estimate | Upper 95% CI | Data reported in: |
| (Intercept) | 0.798 | 0.864 | 0.930 | |
| Visit_two | 0.026 | 0.037 | 0.048 | |
| Visit_three | 0.016 | 0.028 | 0.040 | |
| Visit_four | 0.023 | 0.036 | 0.048 | |
| Visit_five | 0.011 | 0.025 | 0.038 | |
| Visit_six | 0.022 | 0.036 | 0.050 | |
| Moderate/severe exacerbation | –0.036 | –0.024 | –0.012 | Figure 2a |
| Previous moderate/severe exacerbation | –0.012 | –0.008 | –0.005 | Figure 2a |
| Age (years) | –0.0002 | 0.0004 | 0.0011 | |
| Male | –0.020 | –0.010 | 0.000 | |
| US & Canada | –0.006 | 0.006 | 0.017 | |
| Australia & New Zealand & South Africa | 0.015 | 0.041 | 0.067 | |
| Mexico & Central America & South America | –0.041 | 0.062 | 0.165 | |
| LOG eosinophil count | –0.006 | 0.002 | 0.011 | |
| Number of exacerbations previous year | –0.008 | –0.002 | 0.004 | |
| $FEV_1$ continuous | –0.001 | –0.001 | 0.000 | |
| CAT score | –0.014 | –0.013 | –0.013 | |
| ICS use | –0.014 | –0.003 | 0.008 | |
| Severe exacerbation previous year | –0.024 | –0.012 | 0.001 | |
| $FEV_1$ continuous current | 0.002 | 0.0023 | 0.003 | Figure 2a |

| | | | | |
|---|---|---|---|---|
| Visit_two: 160 µg BGF | –0.022 | –0.007 | 0.008 | |
| Visit_two: 320 µg BGF | –0.021 | –0.006 | 0.010 | |
| Visit_two: GFF | –0.016 | –0.001 | 0.015 | |
| Visit_three: 160 µg BGF | –0.011 | 0.005 | 0.021 | |
| Visit_three: 320 µg BGF | –0.009 | 0.007 | 0.023 | |
| Visit_three: GFF | –0.007 | 0.010 | 0.026 | |
| Visit_four: 160 µg BGF | –0.013 | 0.004 | 0.020 | |
| Visit_four: 320 µg BGF | –0.013 | 0.004 | 0.021 | |
| Visit_four: GFF | –0.007 | 0.010 | 0.027 | |
| Visit_five: 160 µg BGF | –0.005 | 0.012 | 0.030 | |
| Visit_five: 320 µg BGF | –0.015 | 0.003 | 0.020 | |
| Visit_five: GFF | –0.009 | 0.009 | 0.028 | |
| Visit_six: 160 µg BGF | –0.017 | 0.002 | 0.020 | |
| Visit_six: 320 µg BGF | –0.024 | –0.005 | 0.014 | |
| Visit_six: GFF | –0.019 | 0.000 | 0.019 | |
| $FEV_1$ continuous current is the current $FEV_1$ percent predicted continuous. | | | | |

| Model 4a[a,c,d] | | | | |
|---|---|---|---|---|
| | Lower 95% CI | Estimate | Upper 95% CI | Data reported in: |
| (Intercept) | 0.880 | 0.945 | 1.010 | |
| Visit_two | 0.028 | 0.040 | 0.051 | |
| Visit_three | 0.020 | 0.031 | 0.043 | |
| Visit_four | 0.026 | 0.039 | 0.051 | |
| Visit_five | 0.014 | 0.028 | 0.041 | |
| Visit_six | 0.025 | 0.039 | 0.053 | |
| Moderate/severe exacerbation | –0.038 | –0.027 | –0.015 | Figure 2b |
| Previous moderate/severe exacerbation | –0.012 | –0.009 | –0.005 | Figure 2b |
| Age (years) | –0.0002 | 0.0004 | 0.0011 | |
| Male | –0.021 | –0.011 | –0.001 | |
| US & Canada | –0.005 | 0.006 | 0.018 | |
| Australia & New Zealand & South Africa | 0.013 | 0.039 | 0.065 | |

| | | | | |
|---|---|---|---|---|
| Mexico & Central America & South America | –0.042 | 0.061 | 0.165 | |
| LOG eosinophil count | –0.006 | 0.003 | 0.011 | |
| Number of exacerbations previous year | –0.007 | –0.001 | 0.005 | |
| Severe COPD | –0.013 | –0.001 | 0.011 | |
| Very severe COPD | –0.028 | –0.010 | 0.009 | |
| CAT score | –0.014 | –0.013 | –0.012 | |
| ICS use | –0.014 | –0.003 | 0.008 | |
| Severe exacerbation previous year | –0.025 | –0.013 | 0.000 | |
| Severe COPD current | –0.025 | –0.018 | –0.010 | Figure 2b |
| Very severe COPD current | –0.056 | –0.045 | –0.035 | Figure 2b |
| Visit_two: 160 µg BGF | –0.020 | –0.005 | 0.010 | |
| Visit_two: 320 µg BGF | –0.018 | –0.002 | 0.013 | |
| Visit_two: GFF | –0.014 | 0.001 | 0.016 | |
| Visit_three: 160 µg BGF | –0.010 | 0.006 | 0.022 | |
| Visit_three: 320 µg BGF | –0.007 | 0.010 | 0.026 | |
| Visit_three: GFF | –0.006 | 0.011 | 0.027 | |
| Visit_four: 160 µg BGF | –0.011 | 0.006 | 0.023 | |
| Visit_four: 320 µg BGF | –0.011 | 0.006 | 0.023 | |
| Visit_four: GFF | –0.006 | 0.011 | 0.029 | |
| Visit_five: 160 µg BGF | –0.003 | 0.015 | 0.032 | |
| Visit_five: 320 µg BGF | –0.014 | 0.004 | 0.022 | |
| Visit_five: GFF | –0.009 | 0.010 | 0.028 | |
| Visit_six: 160 µg BGF | –0.015 | 0.003 | 0.021 | |
| Visit_six: 320 µg BGF | –0.022 | –0.003 | 0.016 | |
| Visit_six: GFF | –0.019 | 0.000 | 0.019 | |

| Model 4b[a,c,d] | | | | |
|---|---|---|---|---|
| | Lower 95% CI | Estimate | Upper 95% CI | Data reported in: |
| (Intercept) | 0.917 | 0.973 | 1.029 | |
| Visit_two | 0.003 | 0.013 | 0.023 | |

| | | | | |
|---|---|---|---|---|
| Visit_three | –0.010 | 0.001 | 0.011 | |
| Visit_four | –0.002 | 0.009 | 0.020 | |
| Visit_five | –0.016 | –0.004 | 0.008 | |
| Visit_six | –0.009 | 0.004 | 0.017 | |
| Moderate/severe exacerbation | –0.007 | 0.004 | 0.015 | Results text (as disutility) |
| Previous moderate/severe exacerbation | –0.005 | –0.002 | 0.001 | Results text (as disutility) |
| Age (years) | 0.0000 | 0.0006 | 0.0011 | |
| Male | –0.011 | –0.003 | 0.006 | |
| US & Canada | –0.004 | 0.005 | 0.015 | |
| Australia & New Zealand & South Africa | 0.000 | 0.021 | 0.043 | |
| Mexico & Central America & South America | –0.016 | 0.071 | 0.159 | |
| LOG eosinophil count | –0.005 | 0.001 | 0.008 | |
| Number of exacerbations previous year | –0.004 | 0.001 | 0.006 | |
| Severe COPD | –0.007 | 0.003 | 0.013 | |
| Very severe COPD | –0.011 | 0.005 | 0.020 | |
| CAT score | –0.005 | –0.005 | –0.004 | |
| ICS use | –0.012 | –0.003 | 0.006 | |
| Severe exacerbation previous year | –0.019 | –0.008 | 0.002 | |
| Severe COPD current | –0.011 | –0.004 | 0.003 | Results text (as disutility) |
| Very severe COPD current | –0.022 | –0.012 | –0.002 | Results text (as disutility) |
| SGRQ total | –0.0033 | –0.0026 | –0.0019 | Results text (as disutility) |
| SGRQ total squared | –0.000039 | –0.000032 | –0.000025 | Results text (as disutility) |
| Visit_two: 160 μg BGF | –0.026 | –0.013 | 0.001 | |
| Visit_two: 320 μg BGF | –0.020 | –0.006 | 0.008 | |
| Visit_two: GFF | –0.012 | 0.002 | 0.016 | |
| Visit_three: 160 μg BGF | –0.014 | 0.000 | 0.014 | |
| Visit_three: 320 μg BGF | –0.013 | 0.002 | 0.016 | |
| Visit_three: GFF | –0.003 | 0.011 | 0.026 | |
| Visit_four: 160 μg BGF | –0.015 | –0.001 | 0.014 | |
| Visit_four: 320 μg BGF | –0.017 | –0.002 | 0.012 | |

| | | | | |
|---|---|---|---|---|
| Visit_four: GFF | –0.004 | 0.011 | 0.026 | |
| Visit_five: 160 µg BGF | –0.006 | 0.009 | 0.024 | |
| Visit_five: 320 µg BGF | –0.012 | 0.004 | 0.019 | |
| Visit_five: GFF | –0.005 | 0.011 | 0.027 | |
| Visit_six: 160 µg BGF | –0.016 | 0.000 | 0.016 | |
| Visit_six: 320 µg BGF | –0.023 | –0.006 | 0.010 | |
| Visit_six: GFF | –0.011 | 0.006 | 0.022 | |

[a]Indicators for each post baseline visit (Visit_two, Visit_three, Visit_four, Visit_five, Visit_six); visit:treatment terms model post-baseline treatment effects; the reference treatment group is budesonide/formoterol fumarate dihydrate

[b]All additional covariates to those included in Model 1 are baseline covariates

[c]Western Europe is the reference group for geographical region

[d]Moderate COPD is the reference category for baseline $FEV_1$ percent predicted versus severe COPD and very severe COPD

*160 µg BGF* 160/14.4/10 µg budesonide/glycopyrronium/formoterol fumarate dihydrate, *320 µg BGF* 320/14.4/10 µg budesonide/glycopyrronium/formoterol fumarate dihydrate, *CAT* COPD Assessment Test, *CI* confidence interval, *COPD* chronic obstructive pulmonary disease, $FEV_1$ forced expiratory volume in 1 second, *GFF* 14.4/10 µg glycopyrronium/formoterol fumarate dihydrate, *ICS* inhaled corticosteroid, *SGRQ* St. George's Respiratory Questionnaire

**Online resource 2** Demographics and clinical characteristics of the mITT population from the ETHOS study (N=8509)

| **Variable** | |
|---|---|
| Age, years, mean (SD) | 64.7 (7.6) |
| Female, n (%) | 3428 (40.3) |
| Male, n (%) | 5081 (59.7) |
| Exacerbations during the 12 months prior to screening, n (%) | |
| 1 | 3699 (43.5) |
| ≥2 | 4810 (56.5) |
| COPD severity (based on GOLD $FEV_1$ % predicted)[a] at screening, n (%) | |
| Moderate | 2427 (28.5) |
| Severe | 5151 (60.5) |
| Very severe | 924 (10.9) |
| Missing | 7 (0.1) |
| Post-rescue medication $FEV_1$ % predicted, mean (SD) | 43.4 (10.3) |

[a]Moderate $FEV_1$ % predicted was defined as ≥50% and <80%; severe $FEV_1$ % predicted was defined as ≥30% and <50%; very severe $FEV_1$ % predicted was defined as <30%

*COPD* chronic obstructive pulmonary disease, *$FEV_1$* forced expiratory volume in 1 second, *$FEV_1$ % predicted* forced expiratory volume in 1 second percentage of predicted normal value, *GOLD* Global Initiative for Chronic Obstructive Lung Disease, *mITT* modified intent-to-treat, *SD* standard deviation

**Online resource 3** Sensitivity analysis based on data not missing at random assumption for predictors of EQ-5D-5L based on $FEV_1$ levels and exacerbations (Question 3; Model 5)[a,b]

| **Variable** | **Coefficient (95% CI)** |
|---|---|
| Predicted baseline EQ-5D-5L utility score for patients with currently moderate COPD with no current or previous exacerbation | 0.726 (0.717, 0.735) |
| *Covariate adjustment from baseline (currently moderate COPD severity with no current or previous exacerbation during the ETHOS trial) in EQ-5D-5L utility score* | |
| Severe COPD | –0.015 (–0.023, –0.008)** |
| Very severe COPD | –0.044 (–0.054, –0.033)** |
| Current exacerbation | –0.021 (–0.053, 0.010) |
| Current exacerbation, additional effect if severe COPD | –0.002 (–0.037, 0.033) |
| Current exacerbation, additional effect if very severe COPD | –0.014 (–0.051, 0.024) |
| Previous exacerbations | –0.017 (–0.021, –0.013)** |

Data represent estimates (95% CI)

Wald p-values; **p<0.01

[a]Model includes covariates as described in Table 1

[b]Based on 3316 patients

*CI* confidence interval, *EQ-5D-5L* EuroQoL 5-dimension 5-level, *$FEV_1$* forced expiratory volume in 1 second
